# *S. pseudintermedius* and *S. aureus* lineages with transmission ability circulate as causative agents of infections in pets for years

**DOI:** 10.1186/s12917-020-02726-4

**Published:** 2021-01-21

**Authors:** Laura Ruiz-Ripa, Carmen Simón, Sara Ceballos, Carmelo Ortega, Myriam Zarazaga, Carmen Torres, Elena Gómez-Sanz

**Affiliations:** 1 Área Bioquímica y Biología Molecular, Universidad de La Rioja, Logroño, 26006 Spain; 2 Departamento de Patología Animal, Universidad de Zaragoza, Zaragoza, 50013 Spain; 3 Institute of Food, Nutrition and Health, ETH Zürich, Schmelzbergstrasse 7, LFV B36, 8092 Zurich, Switzerland; 4 Área de Microbiología Molecular, Centro de Investigación Biomédica de La Rioja (CIBIR), Logroño, 26006 Spain

**Keywords:** MRSP, MRSA, MRSP-ST71, MRSA-CC398, Pets, Infection, Tn*559*

## Abstract

**Background:**

*Staphylococcus pseudintermedius* (SP) and *Staphylococcus aureus* (SA) are common colonizers of companion animals, but they are also considered opportunistic pathogens, causing diseases of diverse severity. This study focused on the identification and characterization of 33 coagulase-positive staphylococci isolated from diseased pets (28 dogs and five cats) during 2009–2011 in a veterinary hospital in Spain in order to stablish the circulating lineages and their antimicrobial resistance profile.

**Results:**

Twenty-eight isolates were identified as SP and five as SA. Nine methicillin-resistant (MR) isolates (27%) carrying the *mecA* gene were detected (eight MRSP and one MRSA). The 55% of SP and SA isolates were multidrug-resistant (MDR). MRSP strains were typed as ST71-*agr*III-SCC*mec*II/III-(PFGE) A (*n*=5), ST68-*agr*IV-SCC*mec*V-B1/B2 (*n*=2), and ST258-*agr*II-SCC*mec*IV-C (*n*=1). SP isolates showed resistance to the following antimicrobials [percentage of resistant isolates/resistance genes]: penicillin [82/*blaZ*], oxacillin [29/*mecA*] erythromycin/clindamycin [43/*erm*(B)], aminoglycosides [18–46/*aacA-aphD, aphA3, aadE*], tetracycline [71/*tet*(M), *tet*(K)], ciprofloxacin [29], chloramphenicol [29/*cat*_pC221_], and trimethoprim-sulfamethoxazole [50/*dfrG*, *dfrK*]. The *dfrK* gene was revealed as part of the *radC*-integrated Tn*559* in two SP isolates. Virulence genes detected among SP isolates were as follow [percentage of isolates]: *siet* [100]*, se-int* [100]*, lukS/F-I* [100]*, sec*_*canine*_ [7], and *expB* [7]. The single MRSA-*mecA* detected was typed as t011-ST398/CC398-*agr*I-SCC*mec*V and was MDR. The methicillin-susceptible SA isolates were typed as t045-ST5/CC5 (*n*=2), t10576-ST1660 (*n*=1), and t005-ST22/CC22 (n=1); the t005-ST22 feline isolate was PVL-positive and the two t045-ST45 isolates were ascribed to Immune Evasion Cluster (IEC) type F. Moreover, the t10576-ST1660 isolate, of potential equine origin, harbored the *lukPQ* and *scn*eq genes. According to animal clinical history and data records, several strains seem to have been acquired from different sources of the hospital environment, while some SA strains appeared to have a human origin.

**Conclusions:**

The frequent detection of MR and MDR isolates among clinical SP and SA strains with noticeable virulence traits is of veterinary concern, implying limited treatment options available. This is the first description of MRSA-ST398 and MRSP-ST68 in pets in Spain, as well the first report of the *dfrK*-carrying Tn*559* in SP. This evidences that current transmissible lineages with mobilizable resistomes have been circulating as causative agents of infections among pets for years.

**Supplementary Information:**

The online version contains supplementary material available at 10.1186/s12917-020-02726-4.

## Background

*Staphylococcus pseudintermedius* (SP) and *Staphylococcus aureus* (SA) are harmless colonizers of the skin and mucosa of humans and animals [1], but they are also frequently implicated in opportunistic infections. In pets, especially in dogs, SP is most frequently detected than SA, both as colonizer and as causative agent of infection. It is recognized as the most common etiological agent implicated in skin and soft tissue infections, otitis, and urinary tract infections in dogs [2–4]. Moreover, SP can also cause diseases in humans, specially associated with dog exposure, which suggests zoonotic transmission [4]. SA is also found in healthy pets in rates between 8 and 12% [5–7].

Similar to methicillin-resistant SA (MRSA) in the clinical setting, methicillin-resistant SP (MRSP) has become a worldwide problem in animal health. It is frequently associated with a multidrug resistance phenotype, which limits the therapeutic options for veterinarians. Moreover, in recent years, several reports have evidenced an increase in the resistance rates for some important antimicrobials, such as fluoroquinolones, in SP isolates recovered from companion animals in European countries [8, 9]. In SP isolates recovered from diseased dogs, previous studies have reported methicillin resistance rates from 10 to 20% [8, 10, 11], although it varies notably depending on the geographic region. However, the methicillin resistance rate increases up to 60% in isolates recovered from canine pyoderma [12]. The spread of MRSP between countries is due to the dissemination of well-known specific genetic lineages such as the clone ST71 in Europe, ST68 in the USA, and ST45/ST112 in Asia [2–4, 10, 13], although all these clones have spread worldwide [4].

Regarding SA, molecular characterization has revealed that companion animals are colonized or infected by hospital-associated (HA) and community-associated (CA) MRSA clones from humans in close contact, which suggests an anthropozoonotic origin [14, 15]. Livestock-associated (LA) MRSA-CC398, which is mainly related with livestock and people with livestock contact, has also been reported causing infections in pets in few occasions [15, 16].

In this study, we identified and performed the molecular characterization of a collection of coagulase-positive staphylococci (CoPS) obtained from diseased pets during a 3-year-sampling period (2009–2011) in the veterinary laboratory at the University of Zaragoza, Spain.

## Results

### Isolates recovered and species identification

Of the 33 CoPS included in this study, 28 were identified as SP and five as SA. The infection site of the animals from which samples were recovered is indicated in Table 1 and Supplementary Table 1. Nine methicillin-resistant isolates carrying the *mecA* gene were detected (eight MRSP and one MRSA), representing 27% of the studied isolates; all of them, except one MRSP isolate, were recovered from dogs. The 55% of SP and SA isolates were multidrug-resistant (MDR) (resistant to at least three families of antimicrobial agents) (Table 1).

**Table 1:** Characterization of the 28 *S. pseudintermedius* and five *S. aureus* isolates recovered from clinical samples of dogs and cats in this study

Strain	Bacterial species	Year	Animal	Type of infection^**a**^	***spa***-MLST/CC-***agr***-SCC***mec***	Antimicrobial resistance phenotype^**b**^	Antimicrobial resistance genotype	Virulence genes detected
C3871^c^	MRSP	2009	Dog	B-J	ST71-*agr*III-SCC*mec*II-III	PEN-OXA-ERY-CLI-GEN-TOB-KAN-STR-TET-CIP^f^-CHL-SXT	*blaZ*, *mecA*, *erm*(B), *aacA/aphD*, *aphA3*, *aadE*, *tet*(K), *cat*_pC221_, *dfrG*	*lukS/F-I, siet, se-int*
C3880	MRSP	2010	Dog	S	ST71-*agr*III-SCC*mec*II-III	PEN-OXA-ERY-CLI-GEN-TOB-STR-TET-CIP^f^-CHL-SXT	*blaZ*, *erm*(B), *aacA/aphD*, *aphA3*, *aadE*, *tet*(K), *cat*_pC221_*, dfrG*	*lukS/F-I, siet, se-int*
C3885	MRSP	2010	Dog	I	ST71-*agr*III-SCC*mec*II-III	PEN-OXA-ERY-CLI-GEN-TOB-KAN-STR-TET-CIP^f^-CHL-SXT	*blaZ*, *erm*(B), *aacA/aphD*, *aphA3*, *aadE*, *tet*(K), c*at*_pC221_, *dfrG*	*lukS/F-I, siet, se-int*
C5355	MRSP	2010	Dog	B-J	ST71-*agr*III-SCC*mec*II-III	PEN-OXA-ERY-CLI-GEN-TOB-KAN-STR-CIP^f^-SXT	*blaZ*, *mecA*, *erm*(B), *aacA/aphD*, *aphA3*, *aadE*, *dfrG*	*lukS/F-I, siet, se-int*
C5613	MRSP	2011	Dog	U-R	ST71-*agr*III-SCC*mec*II-III	PEN-OXA-ERY-CLI-GEN-TOB-KAN-STR-TET-CIP^f^-CHL-SXT	*blaZ*, *mecA*, *erm*(B), *aacA/aphD*, *aphA3*, *aadE*, *tet*(K), *cat*_pC221_, *dfrG*	*lukS/F-I, siet, se-int*
C3866	MRSP	2009	Cat	U-R	ST68-*agr*IV-SCC*mec*V	PEN-OXA-ERY-CLI-KAN-STR-TET-CIP^f^-SXT	*blaZ*, *mecA*, *tet*(M), *erm*(B), *aphA3*, *aadE*, *dfrG*	*lukS/F-I, siet, se-int*
C3870^d^	MRSP	2009	Dog	U-R	ST68-*agr*IV-SCC*mec*V	PEN-OXA-ERY-CLI-KAN-STR-TET-CIP^f^-SXT	*blaZ*, *mecA*, *erm*(B), *aphA3*, *aadE*, *tet*(M), *dfrG*	*lukS/F-I, siet, se-int*
C3869	MRSP	2009	Dog	U-R	ST258-*agr*II-SCC*mec*IV	PEN-OXA-ERY-CLI-KAN-STR-TET-SXT	*blaZ*, *mecA*, *erm*(B), *aphA3*, *aadE*, *tet*(M), *dfrG*	*lukS/F-I, siet, se-int*
C5344	MSSP	2009	Dog	U-R		PEN-ERY-CLI-KAN-STR-TET-CHL-SXT	*blaZ*, *erm*(B), *aphA3*, *aadE*, *tet*(M), *cat*_pC221_, *dfrG*	*lukS/F-I, siet, se-int*
C5345	MSSP	2009	Cat	SI		PEN-ERY-CLI-KAN-STR-TET-CHL-SXT	*blaZ*, *erm*(B), *aphA3*, *aadE*, *tet*(M), *cat*_pC221_, *dfrG*	*lukS/F-I, siet, se-int*
C5360	MSSP	2011	Dog	U-R		PEN-ERY-CLI-KAN-STR-TET-CHL-SXT	*blaZ*, *erm*(B), *aphA3*, *aadE*, *tet*(M), *tet*(K), *dfrG*, *cat*_pC221_	*lukS/F-I, siet, se-int*
C5347	MSSP	2009	Dog	U-R		PEN-ERY-CLI-TET-CHL-SXT	*blaZ*, *erm*(B), *tet*(M), *cat*_pC221_, *dfrG*, *dfrK*^h^	*lukS/F-I, siet, se-int*
C3877	MSSP	2010	Dog	U-R		PEN-KAN-STR-TET	*blaZ*, *aphA3*, *aadE*, *tet*(M)	*lukS/F-I, siet, se-int, expB*
C5351	MSSP	2010	Cat	U-R		PEN-KAN-TET	*blaZ*, *aphA3*, *tet*(M)	*lukS/F-I, siet, se-int*
C3881	MSSP	2010	Dog	U-R		STR-TET-CIP^f^	*str*, t*et*(K), *tet*(M)	*lukS/F-I, siet, se-int*
C5354	MSSP	2010	Dog	U-R		PEN-TET-SXT	*blaZ*, *tet*(M), *dfrG*	*lukS/F-I, siet, se-int*
C5358	MSSP	2011	Dog	U-R		PEN-TET-SXT	*blaZ*, *tet*(M), *dfrG*, *dfrK*^h^	*lukS/F-I, siet, se-int*
C3875	MSSP	2010	Dog	I		PEN-TET	*blaZ*, *tet*(M)	*lukS/F-I, siet, se-int*
C3876	MSSP	2010	Dog	U-R		PEN-TET	*blaZ*, *tet*(M)	*lukS/F-I, siet, se-int, expB*
C5356	MSSP	2011	Dog	U-R		PEN-TET	*blaZ*, *tet*(M)	*lukS/F-I, siet, se-int*
C5362	MSSP	2011	Dog	I		PEN-TET	*blaZ*, *tet*(M)	*lukS/F-I, siet, se-int*
C5353	MSSP	2010	Dog	U-R		TET	*tet*(M)	*lukS/F-I, siet, se-int, sec*_*canine*_
C3873	MSSP	2010	Dog	U-R		PEN	*blaZ*	*lukS/F-I, siet, se-int*
C3878	MSSP	2010	Dog	U-R		PEN	*blaZ*	*lukS/F-I, siet, se-int*
C3879	MSSP	2010	Dog	I		PEN	*blaZ*	*lukS/F-I, siet, se-int, sec*_*canine*_
C5357	MSSP	2011	Dog	U-R		PEN	*blaZ*	*lukS/F-I, siet, se-int*
C3874	MSSP	2010	Dog	I		Susceptible	–	*lukS/F-I, siet, se-int*
C5359	MSSP	2011	Dog	U-R		Susceptible	**–**	*lukS/F-I, siet, se-int*
C3883^e^	MRSA	2010	Dog	S	t011-ST398/CC398-*agr*I-SCC*mec*V	PEN-FOX-ERY-CLI-GEN-TOB-KAN-TET-CIP^g^-SXT	*blaZ*, *mecA*, *erm*(B), *erm*(C), *aacA/aphD*, *tet*(M), *tet*(K), *dfrA*, *dfrG*	*hla*, *hlb*, *hld*, *hlg*
C5612	MSSA	2009	Dog	U-R	t10576-ST1660-*agr*II	PEN	*blaZ*	*lukPQ*, *scn*eq, *hla*, *hlb*, *hld*, *hlg*_v_
C5650	MSSA	2009	Cat	R	t005-ST22/CC22-*agr*I	PEN	*blaZ*	*lukS/F*-PV, *hla*, *hlb*, *hld*, *hlg*
C5610	MSSA	2011	Dog	U-R	t045-ST5/CC5-*agr*II	PEN	*blaZ*	IEC type F, *lukED*, *hla*, *hld*, *hlg*_v_
C5609	MSSA	2011	Cat	I	t045-ST5/CC5-*agr*II	Susceptible	**–**	IEC type F, *lukED*, *hla*, *hld*, *hlg*_v_

^a^B-J, bones-joints infection; U-R, urinary-reproductive infection; R, respiratory infection; I, integumentary infection; S, surgical infection; SI, septic infection

^b^PEN, penicillin; OXA, oxacillin; FOX, cefoxitin; ERY, erythromycin; CLI, clindamycin; GEN, gentamicin; TOB, tobramycin; KAN, kanamycin; STR, streptomycin; TET, tetracycline; CIP, ciprofloxacin; CHL, chloramphenicol; SXT, trimethoprim-sulfamethoxazole

^c^Isolated again one year later of the first recovery

^d^Isolated again two months later of the first recovery

^e^Isolated again three months later of the first recovery

^f^Amino acid change S84L and S80I in GyrA and GrlA proteins, respectively

^g^Amino acid change S84L and S80F in GyrA and GrlA proteins, respectively

^h^The *dfrK* gene was located within the *radC*-integrated Tn*559*

Remarkably, persistent MRSP or MRSA carriage, based on the molecular characteristics of recovered isolates, was detected in three animals evaluated in subsequent samplings. Strains involved were MRSP ST68 clone C3870 (2 months later), MRSP ST71 clone C3871 (1 year later) and MRSA ST398 clone C3883 (after 3 months). Nonetheless, only the initial isolate per animal was included in this study.

### Characterization of SP isolates

Five of the eight MRSP, recovered from diverse infection types, were typed as ST71, *agr*-III, harbored the Staphylococcal Cassette Chromosome *mec *(SCC*me****c***) II-III, and belonged to the same clone (A). Two MRSP recovered from urinary-reproductive infections were typed as ST68, *agr*-IV, SCC*mec* V, and represented two different subclones (B1 and B2). The remaining MRSP isolate, also from a urinary infection, was typed as ST258, *agr*-II, SCC*mec* IV, and displayed pulsed-field gel electrophoresis (PFGE) pattern C.

**Fig. 1:**
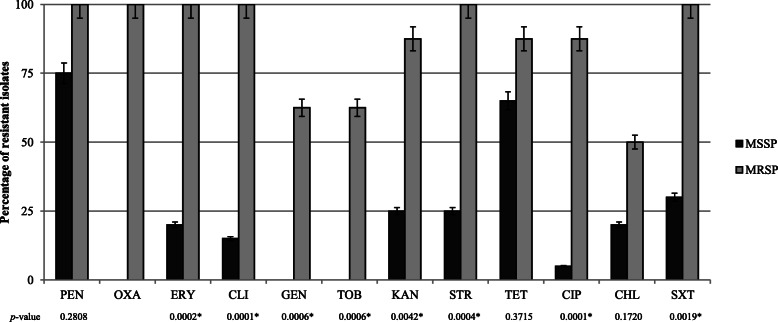
Antimicrobial resistance rate of MSSP and MRSP investigated in this study. PEN, penicillin; ERY, erythromycin; CLI, clindamycin; GEN, gentamicin; TOB, tobramycin; KAN, kanamycin; STR, streptomycin; TET, tetracycline; CIP, ciprofloxacin; CHL, chloramphenicol; SXT, trimethoprim-sulfamethoxazole. The *p*-value (Fisher’s Exact test) is shown below the figure. Asterisks indicate the antimicrobial agents for which statistical differences were found between the antimicrobial resistance rates of MSSP and MRSP (P < 0.05)

The antimicrobial resistance rates of the methicillin-susceptible SP (MSSP) and MRSP isolates, are shown in Fig. 1. Resistance to erythromycin, clindamycin, kanamycin, streptomycin, ciprofloxacin, and trimethoprim-sulfamethoxazole were significantly higher among MRSP isolates. Moreover, resistance to gentamicin and tobramycin were solely detected in MRSP isolates (Fig. 1). Eight out of 28 SP isolates were MRSP (29%). Nine MSSP and all MRSP isolates were MDR, while two MSSP were susceptible to all antimicrobial agents evaluated. The *blaZ* and/or *mecA* resistance genes were responsible of β-lactam resistance in SP isolates. Macrolide and lincosamide resistance was mediated by the *erm*(B) gene in all cases, and aminoglycoside resistance by different combinations of *aacA*/*aphD*, *aphA3*, and *aadE* resistance genes. The *tet*(M) and/or *tet*(K) genes mediated tetracycline resistance. The eight isolates exhibiting chloramphenicol resistance harbored the *cat*_pC221_ gene, while the *dfrG* gene was detected in the 14 isolates that displayed resistance to trimethoprim–sulfamethoxazole, in combination with *dfrK* in two strains (C5358, C5347) (Table 1). The amino acid changes S84L and S80I in the genes encoding the GyrA and GrlA proteins, respectively, were detected in the eight ciprofloxacin-resistant SP isolates. None of the SP isolates showed resistance to vancomycin, linezolid, or fusidic acid.

The virulence genes detected among SP isolates were as follows: *siet* (100%)*, se-int* (100%)*, lukS/F-I* (100%)*, sec*_*canine*_ (7%), and *expB* (7%).

### Characterization of SA isolates

The single MRSA isolate detected was typed as t011-ST398/CC398-*agr*I-SCC*mec*V and was recovered from a surgical infection of a dog. This ST398 strain was resistant to β-lactams, macrolides and lincosamides, aminoglycosides, tetracycline, ciprofloxacin, and trimethoprim-sulfamethoxazole, and carried the *blaZ*, *mecA*, *erm*(B), *erm*(C), *aacA*/*aphD*, *tet*(K), *tet*(M), *dfrA*, and *dfrG* resistance genes. Moreover, the amino acid changes S84L and S80F in the deduced sequences of GyrA and GrlA proteins, respectively, were detected. The MRSA isolate harbored the haemolysins *hla*, *hlb*, *hld*, and *hlg* (Table 1).

The methicillin-susceptible SA (MSSA) isolates were assigned to t045-ST5/CC5 (*n*=2), t10576-ST1660 (*n*=1), and t005-ST22/CC22 (n=1). One MSSA isolate was susceptible to all antimicrobial agents evaluated, and the remaining three only showed penicillin resistance and carried the *blaZ* gene. The MSSA t005-ST22 feline isolate harbored the genes enconding the Panton-Valentine leukocidin (PVL) and the two MSSA t045-ST45 carried the *scn*, *chp*, *sak*, and *sep* genes and, therefore, were ascribed to Immune Evasion Cluster (IEC) type F. Interestingly, the t10576-ST1660 isolate carried the equid-adapted leukocidin *lukPQ* and the equine variant of Staphylococcal Complement Inhibitor (SCIN). Different combinations of haemolysins, encoded by *hla*, *hlb*, *hld*, *hlg*, and *hlg*_v_, were detected among MSSA isolates (Table 1).

### Genetic environment of the *dfrK* gene

**Fig. 2:**
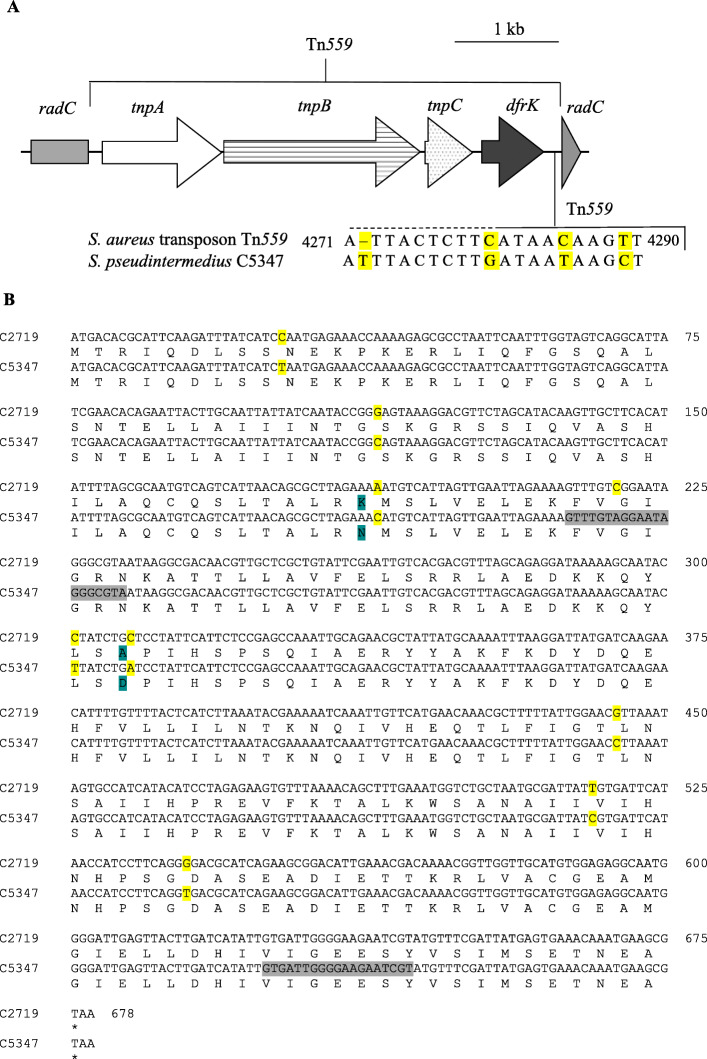
**a** Graphical representation of the Tn*559* structure containing the *dfrK* gene integrated in the chromosomal *radC* gene of the isolate *Staphylococcus pseudintermedius* C5347 (GenBank accession number MT252966) and nucleotide substitutions detected in the non-coding region downstream the *dfrK* gene compared to *Staphylococcus aureus* transposon Tn*559* (GenBank accession number FN677369). The nucleotide positions are set based on the whole Tn*559*. Nucleotide insertions and substitutions are colored in yellow. **b** Amino acid sequence alignment of *radC* gene and resultant RadC of *Staphylococcus pseudintermedius* strain C2719 (GenBank accession number HF679552), where transposon Tn*558* was integrated, that of *S. pseudintermedius* C5347 (GenBank accession number MT252966). The position of the primer pair employed is indicated in grey. Nucleotide substitutions are colored in yellow and amino acid substitutions in blue

The Tn*559*-specific PCRs revealed that the MSSP C5358 harbored a complete Tn*559*, which was integrated in the chromosomal *radC* gene. Detection of the *radC* gene and the Tn*559*-*radC* linkage was not possible for strain MSSP C5347. The whole genome sequencing (WGS) analysis of this strain enabled the identification of the complete Tn*559* and its integration position (Fig. 2) (GenBank accession number MT252966). Nucleotide and amino acid sequence alignment of Tn*559* C5347 with reference *Staphylococcus aureus* transposon Tn*559* (GenBank accession number FN677369) revealed the insertion of one nucleotide and three nucleotide substitutions in the non-coding region downstream the *dfrK* gene (Fig. 2). Remarkably, a single point mutation in the hybridization sequence of the *radC* forward primer was detected (radC-fw: 5′-GTC/AGGAATAGGGCGTA-3′), which resulted responsible for the absence of PCR amplification. Moreover, nucleotide and amino acid alignment with the *radC* gene of *S. pseudintermedius* strain C2719, harboring transposon Tn*558* (GenBank accession number HF679552), revealed the presence of seven synonymous point mutations (C27T, G114C, C219A, C301T, G444C, T516C, G540T) plus two additional non-synonymous substitutions in the deduced RadC sequence (K63N, A103D) (Fig. 2). Tn*559* circular intermediates were detected.

## Discussion

This is the first description of MRSA-ST398 and MRSP-ST68 in pets in Spain, as well the first report of the *dfrK*-carrying Tn*559* in SP. This evidences that current transmissible lineages with mobilizable resistomes have circulated unnoticeably in Spain as causative agents of infections in pets for years.

In this study, 28 of the 33 CoPS from diseased pets were identified as SP, which was expected since this is the most common staphylococcal species detected both as colonizer and cause of infection in companion animals, specially dogs [4, 5]. In this work, SP and SA were detected causing infection in three and two cats, respectively. These species have been formerly recovered from diseased cats [1, 17], however, a recent study has reported predominance of other staphylococcal species, such as *S. felis* and *S. haemolyticus*, among feline infections [1].

Five of the eight MRSP isolates detected in this study were ascribed to the genetic lineage ST71 and showed the same PFGE pattern. They were isolated from five different dogs in different years and there was no apparent relation among these animals; however, all of them underwent surgery in the veterinary hospital. Regardless they were assisted by different veterinarians, and the operating room and/or surgery material are recurrently disinfected, none of them can be excluded as potential infection sources. MRSP-ST71 is the major clone in Europe [2, 3], and there are already few descriptions of this genetic lineage in healthy dogs in Spain [18]. However, recent studies have reported a downward trend in the prevalence of the MRSP-ST71 lineage among companion animals in northern Europe [11, 19] and France [3]. The MRSP-ST258 lineage, which was detected in this study in 2009, seems to be replacing MRSP-ST71 in these countries [11, 19]. The remaining two MRSP detected in this study belonged to the ST68, a genetic lineage known to be predominant in USA, although it has been detected outside this country [4]. However, to the best of our knowledge, this is the first description of MRSP-ST68 isolated from companion animals in Spain.

Former studies reported MRSA belonging to the HA-MRSA and CA-MRSA lineages among isolates recovered from companion animals [9, 15]; however, in this work, the only MRSA isolate detected belonged to the LA-MRSA CC398 genetic lineage. Moreover, this isolate lacked the genes of the IEC system, which suggests an animal origin. LA-MRSA CC398 recovered from diseased dogs has been reported in few occasions [9, 15, 16], however, to our knowledge, this is the first description in Spain. Regarding MSSA, isolates belonging to CC22 and CC5 are clonal groups widespread among companion animals [9, 15, 20]. The remaining MSSA isolate was typed as t10576-ST1660, and, interestingly, the single description of this genetic lineage is in a contemporary isolate (obtained in the same week) recovered from one hospitalized equine in the same veterinary hospital, which displayed the same antimicrobial resistance pheno/genotype and virulence gene content [21]. Moreover, the SA t10576-ST1660 isolate recovered in this study harbored the leukocidin *lukPQ* and the *scn*eq, which are equid-adapted virulence factors [22, 23]. This supports the hypothesis of a plausible equine origin and suggests the transmission of the MSSA strain between animal species in the veterinary hospital.

The frequency of methicillin resistance and MDR isolates among CoPS recovered from pets in this study represents a great concern for veterinary medicine. This is also a public health problem due to the exposure and interspecies transmission of SA/SP among pets and owners [6, 24], as well as potential transference of resistance genes to human-adapted staphylococcal strains. The rate of methicillin resistance observed in this study (27%) is higher than the one determined among clinical isolates from companion animals in several countries, such as Australia (12%) [10, 17], Finland (14%) [19], the Netherlands [11], and France [8]. As in this report, MRSP-ST71 are often MDR [2, 11], and they are more likely to display fluoroquinolone resistance than other STs [10, 11]. Fortunately, in line with former reports, all isolates recovered here, both SA and SP, were susceptible to important or last resort antimicrobials in human medicine, such as vancomycin and linezolid [2, 9].

All SP isolates, both MSSP and MRSP, carried the virulence determinants *lukS/F*-I, *siet*, and *se-int*, which have been previously detected in both commensal and clinical SP isolates, suggesting they may be ubiquitous in this staphylococcal species [5, 18, 20]. One MSSA t005-ST22/CC2 recovered from a respiratory infection of a cat was PVL-positive. The PVL is one of the most important virulence determinants produced by SA, which has a critical role in the pathogenesis of skin and soft tissue infections. CC22 is an important genetic lineage of PVL-positive MSSA also implicated in hospital outbreaks [25, 26]. Moreover, the two MSSA t045-ST5/CC5 isolates carried the human-adapted *scn*, *chp*, *sak*, and *sep* genes of the IEC system, which suggests a human-to-animal transmission. To this regard, former studies have determined the interspecies transmission ability of SA from humans to pets [6, 24], which represents a source for further transmission and a risk for infection.

The *dfrK* gene has been rarely detected in SP isolates [9, 27]. Indeed, the *dfrK*-carrying transposon Tn*559* was firstly reported in a porcine MSSA ST398 isolate [28] and thereafter in SA belonging to the same genetic lineage [21], but also in *Enterococcus faecium* [29]. However, to the best of our knowledge, we report here the first description of the Tn*559* in SP. This could suggest an exchange of resistance genes between staphylococci and other Gram-positive bacteria, including enterococci, of animal but also from human origin.

## Conclusions

Presently relevant MRSP and MRSA genetic lineages with noticeable virulence traits were detected in isolates recovered during 2009–2011, including the first description of MRSA ST398 and MRSP ST68 in pets in Spain. In addition, the apparent dissemination of CoPS strains in the veterinary hospital highlights the importance of further investigating SA and SP sources and survival ability as contaminants, their population structure and epidemiology, as well as their antimicrobial resistance pattern and transmission ability.

## Methods

### Study population

A total of 33 CoPS isolates obtained from 28 and five diseased dogs and cats (one isolate/animal), respectively, were obtained in the Veterinary Laboratory of the University of Zaragoza (Zaragoza, Spain) during the years 2009 (9 isolates), 2010 (15), and 2011 (9), and were included in this study. The samples were taken from the infection site (Supplementary Table S1). The isolates were stored frozen at − 80 °C until they were studied.

### Isolation and identification of SP and SA isolates

Identification of isolates was performed by biochemical assays, including colony morphology, Gram staining, catalase and DNase activities, and API20-STAPH. The identification of SA and SP was determined by a multiplex PCR that amplifies the specific *nuc* gene of SA or *S. intermedius*/SP [30]. Discrimination between *S. intermedius* and SP was carried out by digestion of the *pta* gene amplicon with *Mbo*I enzyme [31].

### Molecular typing and clonal relatedness

All SA were subjected to *spa-*typing by PCR and amplicon sequencing, and the obtained sequences were analyzed using Ridom Staph-Type software version 1.5.21 (Ridom GmbH, Münster, Germany) [32]. Multi Locus Sequence Typing (MLST) was performed in all SA isolates [32], and according to the sequence-type (ST), the isolates were ascribed to the different clonal complexes (CC). MLST of SP isolates was likewise performed as previously described [33]. All isolates were characterized by *agr*-typing following standard methodology [32, 33]. SCC*mec*-typing was undergone in MRSP and MRSA as previously described [13, 34].

PFGE of total DNA restricted with *Sma*I enzyme was performed on MRSP as previously described [35]. Isolates were considered different clones when they exhibited more than three bands of difference in PFGE band patterns and subclones when PFGE band patterns differed between 1 and 3 bands [36].

### Antimicrobial resistance phenotype and genotype

The susceptibility to 17 antimicrobials was determined by agar disk-diffusion method. The antimicrobial agents tested were as follows (μg/disk): penicillin (10 units), oxacillin (1), cefoxitin (30) erythromycin (15), clindamycin (2), gentamicin (10), tobramycin (10), kanamycin (30), streptomycin (10), tetracycline (30), ciprofloxacin (5), mupirocin (200), vancomycin (30), chloramphenicol (30), linezolid (30), fusidic acid (10), and trimethoprim-sulfamethoxazole (1.25+ 23.75). The CLSI guidelines [37] was used for all antimicrobials, except for streptomycin, mupirocin, and fusidic acid, for which the methods and breakpoints recommended by the Société Française de Microbiologie [38] were employed.

The presence of 34 antimicrobial resistance genes was investigated by PCR: *mec*A*, blaZ, erm*(A), *erm*(B), *erm*(C), *erm*(T), *mph*(C), *msr*(A), *msr*(B), *lnu*(A), *vga*(A), *vga*(C), *aacA-aphD, aphA3, aadE*, *aadD*, *aadA, str*, *tet*(K), *tet*(M), *tet*(L), *sat4* (even though streptothricin susceptibility was not tested), *fexA, fexB, cfr, optrA, poxtA, cat*_ps194_*, cat*_pC221_*, cat*_pC223_, *dfrA*, *dfrD*, *dfrG*, and *dfr*K [18, 39, 40]. Positive controls from the collection of the University of La Rioja were included in all PCR assays.

Mutations in the genes encoding the GyrA and GrlA proteins were investigated in ciprofloxacin-resistant SA and SP isolates by PCR and sequencing [18, 41]. The corresponding sequences of *S. aureus* NCTC 8325 (GenBank accession number CP000253) and *S. pseudintermedius* KM1381 (GenBank accession number AM262969 and AM262972) were used as references.

### Detection of virulence genes

The presence of the leukocidin genes *lukSF*-PV, *lukM, lukED*, and *lukPQ* was investigated in all SA isolates [21, 23]. They were also screened for the presence of haemolysin genes (*hla*, *hlb*, *hld*, *hlg*, and *hlg*_v_), exfoliative genes (*eta*, *etb*, and *etd*), and the toxic shock syndrome toxin-1 (*tst*) [21]. PCR-based determination of the five genes (*scn*, *chp*, *sak*, *sea,* and *sep*) that comprises the IEC system as well the equine variant of SCIN encoded by *scn*eq were likewise investigated [32, 42]. In addition, SP isolates were screened for the presence of the leukocidin gene *lukS/F-I*, the exfoliative genes *siet*, *expA*, and *expB*, and the enterotoxin genes *si-ent* and *sec*_*canine*_ by PCR [20].

### Statistical analysis

Potential statistical differences between the antimicrobial resistance rates in MRSP and MSSP isolates were compared using the Fisher’s Exact test with the R Commander program. *P* < 0.05 was considered a statistically significant result.

### Genetic environment of the *dfrK* gene in *S. pseudintermedius* isolates

The possibility that the *dfrK* gene was located within the Tn*559* and integrated within the chromosomal *radC* gene was investigated by specific PCRs targeting the different constituents of Tn*559* and their physical linkage to *radC*, as previously described [21, 43]. Strains negative for at least one primer combination were submitted to WGS.

### Whole genome sequencing of Tn*559* carrying strain negative for *radC* integration

WGS was performed on SP strain C5347 using PacBio Sequel and Illumina Miseq 2 × 300 bp platforms prior phenol-chloroform DNA extraction, as previously described [44]. Raw PacBio reads were assembled using Canu [45] with default parameters and setting an estimated genome size of 3 Mb. The resulting assembled contigs were then polished as follows: First, Illumina raw reads were quality-trimmed using Trimmomatic [46] and aligned against the assembled PacBio contigs using Bowtie2 [47]. Then, the resulting bam files were used to fix individual base errors, indels and local missassemblies using Pilon [48].

Resulting genes on the assembled contigs were predicted using Prodigal [49]. tRNA and rRNA genes were predicted using tRNAscan-SE [50], ssu-align [51] and meta-rna [52]. Predicted protein sequences were compared against the NCBI nr database using DIAMOND [53], and against COG [54] and TIGFRAM [55] using HMMscan [56] for taxonomic and functional annotation.

## Supplementary Information

**Additional file 1: Table S1.** Type of infection, sample and sampling method used to obtain the thirty-three coagulase-positive staphylococci of this study.

## Data Availability

The entire transposon Tn*559* plus the truncated *radC* gene of *S. pseudintermedius* C5347, comprising 5′069 bps, have been deposited in the Genbank database with accession number MT252966.

## Abbreviations

SP: *Staphylococcus pseudintermedius*
SA: *Staphylococcus aureus*
MR: methicillin-resistant
MDR: multidrug-resistant
SCC*mec*: Staphylococcal cassette chromosome
ST: sequence type
CC: clonal complex
PVL: Panton-Valentine leukocidin
IEC: immune evasion cluster
MRSA: methicillin-resistant *Staphylococcus aureus*
MRSP: methicillin-resistant *Staphylococcus pseudintermedius*
HA: hospital-associated
CA: community-associated
LA: livestock-associated
CoPS: coagulase-positive staphylococci
PFGE: pulsed-field gel electrophoresis
MSSP: methicillin-susceptible *S. pseudintermedius*
MSSA: methicillin-susceptible *S. aureus*
SCIN: staphylococcal complement inhibitor
MLST: multilocus sequence typing
CLSI: clinical laboratory standards institute
PCR: polymerase chain reaction
WGS: whole genome sequencing
