# Supplementary material for: S. pseudintermedius and S. aureus lineages with transmission ability circulate as causative agents of infections in pets for years
Source: BMC Vet Res. 2021 Jan 21;17:42. doi: 10.1186/s12917-020-02726-4 (PMC7819200; doi:10.1186/s12917-020-02726-4)
Supplement: Supplementary file 1 — Additional file 1: Table S1. Type of infection, sample and sampling method used to obtain the thirty-three coagulase-positive staphylococci of this study. [file 12917_2020_2726_MOESM1_ESM.docx]

***S. pseudintermedius* and *S. aureus* lineages with transmission ability circulate as causative agents of infections in pets for years**

Laura Ruiz-Ripa, Carmen Simon, Sara Ceballos, Carmelo Ortega, Myriam Zarazaga, Carmen Torres, Elena Gómez-Sanz*

***Corresponding author:**

Elena Gómez-Sanz, Ph.D.

ETH Zurich

Institute of Food, Nutrition and Health (IFNH)

Laboratory of Food Microbiology

Schmelzbergstrasse 7, LFV B36

8092 Zurich

Switzerland

Office: [(+41) 44 632 7094](tel:%28%2B41%29%2044%20632%203335)

eMail: [elena.gomez@hest.ethz.ch](mailto:elena.gomez@hest.ethz.ch)

**Table S1**. Type of infection, sample and sampling method used to obtain the thirty-three coagulase-positive staphylococci of this study.

| **Strain** | **Bacterial species** | **Type of infection^a^** | **Sample** | **Sampling method** |
| --- | --- | --- | --- | --- |
| C3871 | MRSP | B-J | Muscular swab | Disinfection and sterile swab |
| C3880 | MRSP | S | Wound drainage | Disinfection and sterile swab |
| C3885 | MRSP | I | Seroma | Syringe |
| C5355 | MRSP | B-J | Joint swab | Syringe |
| C5613 | MRSP | U-R | Mammary secretion | Disinfection and sterile swab |
| C3866 | MRSP | U-R | Urine | Cystocentesis |
| C3870 | MRSP | U-R | Vaginal swab | Disinfection and sterile swab |
| C3869 | MRSP | U-R | Urine | Cystocentesis |
| C5344 | MSSP | U-R | Urine | Cystocentesis |
| C5345 | MSSP | SI | Pleural effusion | Syringe |
| C5360 | MSSP | U-R | Urine | Cystocentesis |
| C5347 | MSSP | U-R | Urine | Cystocentesis |
| C3877 | MSSP | U-R | Urine | Cystocentesis |
| C5351 | MSSP | U-R | Urine | Cystocentesis |
| C3881 | MSSP | U-R | Urine | Cystocentesis |
| C5354 | MSSP | U-R | Urethral swab | Disinfection and sterile swab |
| C5358 | MSSP | U-R | Urine | Cystocentesis |
| C3875 | MSSP | I | Ear swab | Disinfection and sterile swab |
| C3876 | MSSP | U-R | Vaginal swab | Disinfection and sterile swab |
| C5356 | MSSP | U-R | Urine | Cystocentesis |
| C5362 | MSSP | I | Ear swab | Disinfection and sterile swab |
| C5353 | MSSP | U-R | Vaginal swab | Disinfection and sterile swab |
| C3873 | MSSP | U-R | Urine | Cystocentesis |
| C3878 | MSSP | U-R | Vaginal swab | Disinfection and sterile swab |
| C3879 | MSSP | I | Ear swab | Disinfection and sterile swab |
| C5357 | MSSP | U-R | Vaginal swab | Disinfection and sterile swab |
| C3874 | MSSP | I | Ear swab | Disinfection and sterile swab |
| C5359 | MSSP | U-R | Mammary secretion | Disinfection and sterile swab |
| C3883 | MRSA | S | Post-surgical fistula | Disinfection and sterile swab |
| C5612 | MSSA | U-R | Mammary secretion | Disinfection and sterile swab |
| C5650 | MSSA | R | Nasal-maxillary biopsy | Biopsy |
| C5610 | MSSA | U-R | Urine | Cystocentesis |
| C5609 | MSSA | I | Abscess swab | Disinfection and sterile swab |

^a^B-J, bones-joints infection; U-R, urinary-reproductive infection; R, respiratory infection; I, integumentary infection; S, surgical infection; SI, septic infection.
